# Pancreatic stent migration-induced jejunal perforation following pancreaticoduodenectomy: case report and literature review

**DOI:** 10.1186/s12893-026-04120-7

**Published:** 2026-08-12

**Authors:** Luobin Han, Manman He, Xiaohui Liu, Changjing Wang, Buyun Song

**Affiliations:** 1 Chengde Medical University, Chengde, Hebei 067000 China; 2 Department of Anesthesiology, Handan Central Hospital, Handan, Hebei 056000 China; 3 Department of General Surgery II, Handan Central Hospital, Handan, Hebei 056000 China

**Keywords:** Pancreaticoduodenectomy, Pancreatic duct stents, Postoperative pancreatic fistula (POPF), Perforation, Whipple procedure

## Abstract

**Background:**

Jejunal perforation resulting from pancreatic stent migration represents a rare but potentially lethal complication after pancreaticoduodenectomy (PD), characterized by non-specific clinical manifestations. Clinicians must pay close attention to this postoperative adverse event. Standardized and precise intraoperative stent placement, combined with intensive postoperative surveillance and periodic follow-up, is essential to enable timely clinical intervention.

**Case presentation:**

Two patients treated at our center underwent laparoscopic pancreaticoduodenectomy (LPD) for cholangiocarcinoma. On postoperative day 3 and day 6 respectively, both patients developed sudden severe abdominal pain along with fever and increased output from abdominal drains. Physical examination showed board-like abdominal rigidity, and imaging studies revealed abdominopelvic fluid collection. Emergency laparotomy confirmed jejunal perforation secondary to migrated pancreatic stents. The redundant segments of the stents were trimmed, followed by repair of the jejunal perforations. Both patients recovered uneventfully and were discharged smoothly.

**Conclusion:**

Perforation secondary to internal pancreatic stents arises from multiple contributing factors. In the early postoperative phase, this complication is difficult to differentiate from anastomotic leakage. Unlike anastomotic leakage, however, stent-induced perforation rarely heals spontaneously, necessitating prompt accurate diagnosis and targeted intervention. For early-onset presentations, aggressive surgical intervention is recommended, whereas endoscopic management constitutes the first-line strategy for late-onset cases, with surgical repair undertaken as needed.

**Supplementary Information:**

The online version contains supplementary material available at https://doi.org/10.1186/s12893-026-04120-7.

## Introduction

LPD serves as the standard surgical procedure for benign and malignant lesions involving the pancreatic head, distal common bile duct, descending duodenum, and adjacent regions [1]. Pancreatic fistula represents the most prevalent and severe complication of this operation [1, 2]. Placement of pancreatic duct stents following pancreaticojejunostomy is mainly intended to lower the incidence of postoperative pancreatic fistula and anastomotic stricture [2–5]. Nevertheless, stent migration can be induced by multiple factors, such as an overly long segment of the stent retained within the jejunum and rigid stent material [5–7], Excessive anastomotic tension leads to angulation of the proximal jejunum [7], as well as intestinal wall edema [8, 9]. Subsequent stent migration may induce severe complications including pancreatitis, intestinal obstruction, cholangitis and intestinal perforation [1, 3, 4]. This study retrospectively reviewed two cases of jejunal perforation caused by postoperative pancreatic duct stent migration in our center, combined with a literature review, to raise clinicians’ awareness and vigilance of this complication.

## Case report

### Case 1

**Fig. 1:**
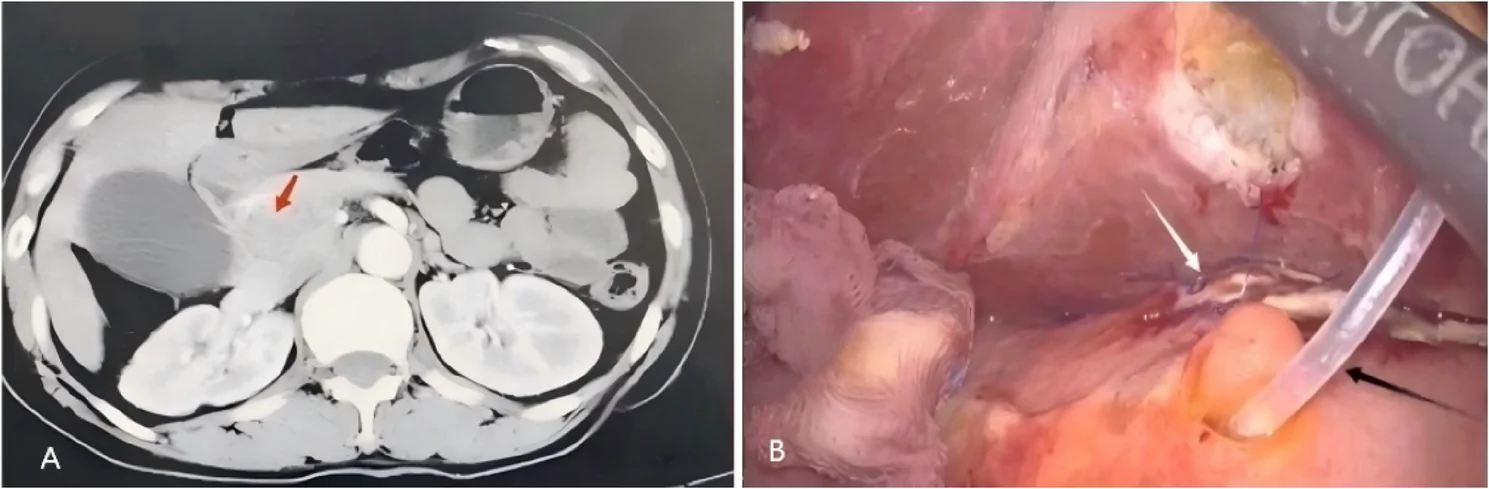
Preoperative contrast-enhanced CT and intraoperative perforation findings of Case 1. **a** Preoperative contrast-enhanced computed tomography (CECT) reveals a tumor in the middle segment of the common bile duct (red arrow). **b** intraoperative imaging shows the stentpenetrating the jejunum (black arrow) and thepancreaticojejunal anastomosis (white arrow)

A woman aged in her fifties was admitted in November 2019 with a one-week history of intermittent epigastric pain accompanied by generalized cutaneous and scleral jaundice. Pre-admission abdominal MRI demonstrated low obstructive jaundice secondary to a space-occupying lesion at the pancreatic head segment of the common bile duct, with diffuse biliary dilatation. The patient was previously healthy with no hereditary family history. Physical examination on admission revealed marked jaundice of the skin and sclera, as well as mild deep tenderness beneath the xiphoid process. Laboratory tests showed elevated levels of bilirubin, alkaline phosphatase, transaminase, CA-199 and CA-50. Contrast-enhanced abdominal CT indicated a malignant mass in the middle common bile duct highly suggestive of carcinoma, with proximal biliary dilatation (Fig. 1A). The preliminary diagnosis was choledochal carcinoma complicated by obstructive jaundice.

The patient underwent elective LPD on December 4, 2019. Digestive tract reconstruction was performed using the Child procedure. Pancreaticojejunostomy was accomplished with the modified Blumgart technique, consisting of outer-layer transpancretic U-sutures combined with inner duct-to-mucosa anastomosis. A 6 Fr, 8 cm small-bore silicone stent was placed to bridge the pancreaticojejunostomy and secured by wrapping one loop of the outer anastomotic suture around the stent before knotting. Hepaticojejunostomy was performed with continuous 4 − 0 absorbable barbed sutures, and an antecolic side-to-side gastrojejunostomy was created using a linear cutting stapler. The hepaticojejunostomy was constructed approximately 6 cm distal to the pancreaticojejunostomy, and the antecolic gastrojejunostomy was performed roughly 50 cm downstream from the biliary anastomosis. The operation proceeded uneventfully, with a total operative duration of 390 min and an estimated intraoperative blood loss of 200 mL. Upon final hemostasis and peritoneal irrigation at the end of surgery, the pancreatic stent was noted to have migrated into the jejunal blind loop and bulged the intestinal wall, with a high risk of impending perforation. A 6-cm mini-laparotomy was subsequently made to retrieve the specimen and reposition the stent into the afferent limb between the pancreaticojejunostomy and hepaticojejunostomy. Three 6 mm×9 mm silicone drainage tubes were placed posterior to the hepaticojejunostomy, as well as anterior and posterior to the pancreaticojejunostomy. Routine postoperative supportive care was administered thereafter.

On the early morning of postoperative day 3, the patient developed sudden severe abdominal pain accompanied by a high fever of 39 °C. Approximately 500 mL of dark green-brown fluid drained from the tube anterior to the pancreaticojejunostomy. Emergency laboratory tests indicated significantly increased inflammatory markers, and abdominal CT revealed extensive abdominopelvic fluid collection. Physical examination demonstrated board-like abdominal rigidity. Considering suspected jejunal perforation secondary to pancreatic stent migration, emergency laparoscopic exploration was performed. Intraoperatively, a perforation was detected on the anterior wall of the jejunal blind loop adjacent to the pancreaticojejunostomy, with approximately 2.5 cm of the silicone stent exposed outside the intestinal lumen. No leakage was observed at any anastomotic site (Fig. 1B). Approximately 3 cm of the redundant stent was trimmed, and the jejunal perforation was repaired primarily. The original anterior pancreatic drainage tube was replaced with a double-lumen irrigation-suction drain. The reoperation was uneventful. Postoperative antibiotic therapy with cefoperazone-sulbactam was administered. The patient recovered smoothly after the second operation and was discharged after a total hospitalization of 30 days.

Postoperative pathological examination revealed a protuberant, moderately differentiated adenocarcinoma of the common bile duct, measuring 3 × 2 × 1.5 cm. The tumor infiltrated the full thickness of the bile duct wall and invaded the surrounding pancreatic tissue. No lymph node metastasis was identified (0/18 resected lymph nodes). During the 48-month follow-up period, the patient remained free of tumor recurrence and metastasis and maintained a good survival status.

### Case 2

**Fig. 2:**
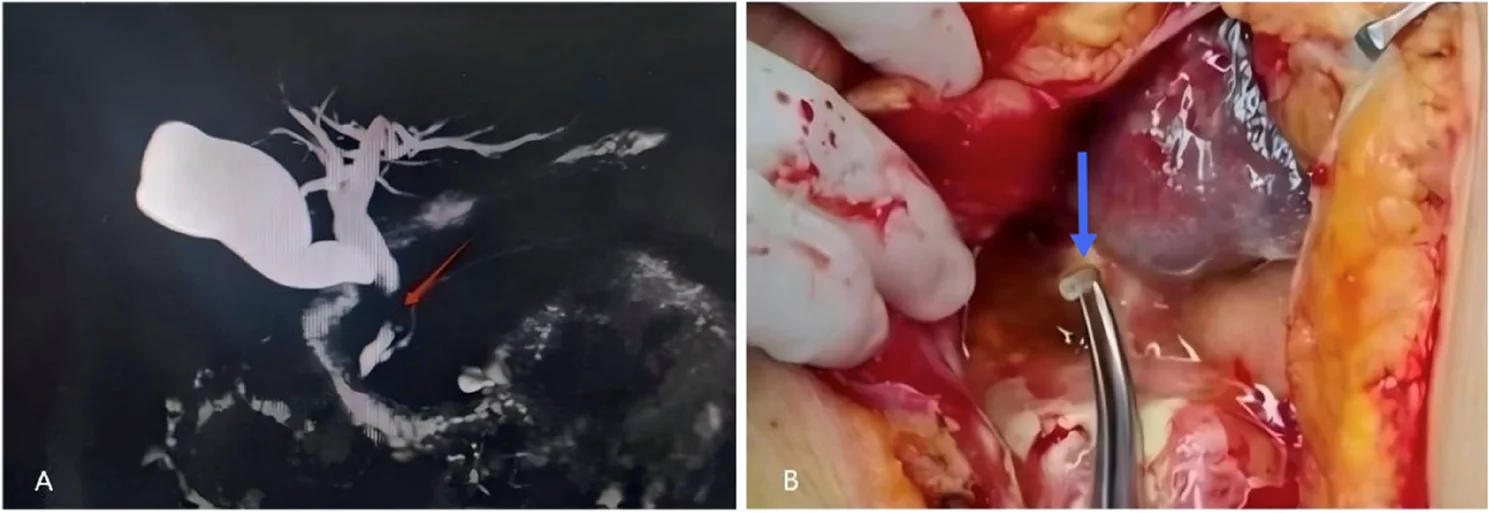
Preoperative MRCP images and intraoperative perforation findings of Case 2. **a** Preoperative three-dimensional magnetic resonance cholangiopancreatography (MRCP) demonstrates a tumor in the distal segment of the common bile duct (red arrow). **b **intraoperative imaging shows the stent penetrating the jejunum (blue arrow)

A 50-year-old male patient was admitted in October 2020 with a one-month history of intermittent chills and fever, accompanied by generalized cutaneous and scleral jaundice for one week. The patient was previously healthy with no hereditary family disease history. On admission physical examination, marked jaundice of the skin and sclera was observed, with remarkable tenderness in the right upper quadrant and a positive Murphy’s sign. Laboratory tests revealed elevated levels of white blood cells, bilirubin, alkaline phosphatase, transaminase, CA-199 and CA-50. Contrast-enhanced abdominal CT indicated low biliary obstruction, raising suspicion of a space-occupying lesion. Abdominal contrast-enhanced MRI showed wall thickening and luminal stenosis of the distal common bile duct, highly suggestive of a space-occupying lesion, along with gallbladder wall thickening consistent with cholecystitis (Fig. 2A). The preliminary diagnoses were as follows: 1.distal common bile duct mass with obstructive jaundice; 2. acute cholangitis; 3.cholecystitis.

The patient underwent ultrasound-guided percutaneous cholecystostomy on October 31, 2020 to relieve biliary obstruction, followed by elective LPD on November 4, 2020. Intraoperatively, the gallbladder drainage catheter was found dislodged into the abdominal cavity, and severe intra-abdominal adhesions secondary to bile leakage were identified. Conversion to open laparotomy was therefore performed. Digestive tract reconstruction was carried out using the same approach as Case 1. A 6 Fr, 10 cm small-bore silicone stent was placed to bridge the pancreaticojejunostomy, and the anastomotic suture was wrapped one full loop around the stent and secured with a knot. Hepaticojejunostomy was constructed approximately 6 cm distal to the pancreaticojejunostomy. The operation proceeded uneventfully with an operative duration of 220 min and an estimated intraoperative blood loss of 100 mL. Standard routine postoperative supportive care was provided.

The patient underwent ultrasound-guided percutaneous cholecystostomy on October 31, 2020 to alleviate biliary obstruction, and elective LPD was performed on November 4, 2020. Intraoperatively, the gallbladder drainage catheter was dislodged into the abdominal cavity, and severe intra-abdominal adhesions resulting from bile leakage were observed, leading to conversion to open laparotomy(Fig. 2B). Digestive tract reconstruction was performed using the identical technique applied in Case 1. A 6 Fr, 10 cm small-bore silicone stent was inserted to bridge the pancreaticojejunostomy, and the hepaticojejunostomy was created approximately 6 cm distal to the pancreaticojejunostomy. The operation proceeded uneventfully, with a total operative time of 220 min and an estimated intraoperative blood loss of 100 mL. Routine postoperative supportive treatment was administered.

Postoperative pathological results showed moderately differentiated adenocarcinoma arising from dysplastic mucosal glands of the distal common bile duct. The tumor measured 1.5 × 1.5 × 0.3 cm and infiltrated the full thickness of the bile duct wall. No metastatic carcinoma was detected in the harvested lymph nodes (0/10). Acute cholecystitis was also confirmed. The patient remained free of tumor recurrence and metastasis with favorable survival during the 37-month follow-up period.

## Discussion

Pancreatic fistula, the most prevalent complication following PD can trigger life-threatening adverse events including intra-abdominal abscess and massive intraperitoneal hemorrhage [10], Intraoperative stent placement is performed to reduce the incidence of pancreatic fistula. Based on drainage patterns, stents are classified into external and internal pancreatic drainage stents, each with distinct advantages and disadvantages. The advantages of external drainage lie in the extraperitoneal diversion of pancreatic juice to relieve anastomotic tension; clinicians can evaluate anastomotic healing and stent patency by observing the volume, color and other characteristics of drained fluid [1]. Its disadvantages include long-term pancreaticojejunostomy stricture, peritubular skin erosion, as well as accidental occlusion or dislodgement of the drainage stent [1, 2]. Internal drainage boasts the merits of simple intraoperative manipulation, physiological drainage pattern, and minimal loss of electrolytes and trypsin [1, 2]. However, stent occlusion or migration may induce complications such as pancreatitis, intestinal obstruction, intestinal perforation and cholangitis [1, 3, 4].

Guo C et al. included seven randomized controlled trials involving a total of 847 patients. The results demonstrated no statistically significant difference in the incidence of postoperative pancreatic fistula between the stenting group and the non-stenting group (RR = 0.85, 95%CI: 0.57–1.26, *P* = 0.41) [11]. Nevertheless, internal stents eliminate the need for external transcutaneous drainage catheters, which can relieve patient discomfort associated with external drainage, shorten hospital stay, and improve patients’ quality of life. Therefore, internal drainage has become one of the most widely adopted strategies in clinical practice [10–12]. Internal drainage stents usually dislodge spontaneously into the small intestine after complete and mature healing of the anastomosis and are eventually excreted with stool [7, 10, 13]. A retrospective study conducted by Kadowaki et al. reported that the median time from stent implantation to spontaneous intestinal passage was 454 days, and the cumulative stent passage rate at 1 year was merely 41% [10]. Stent diameter ≥ 5 Fr, ≥ 5 sutures for pancreatic duct mucosa anastomosis, and grade B/C pancreatic fistula are independent risk factors for delayed stent passage and subsequent long-term complications [10].

A quality control seminar was held postoperatively for Case 1 in our center, and the following intraoperative deficiencies were summarized: (1) To facilitate exposure of the pancreatic duct, the pancreas was transected obliquely upward and leftward on the left side of the superior mesenteric vein-portal vein confluence. After pancreaticojejunostomy, the long axis of the pancreatic duct formed an obtuse angle with the jejunal blind loop and an acute angle with the afferent jejunal limb, allowing the distal end of the stent to perpendicularly protrude into the jejunal blind loop. (2) Although stent migration was identified and the stent was repositioned intraoperatively, the adverse impact of the anatomical angle was neglected, resulting in recurrent stent migration into the jejunal blind loop and subsequent intestinal perforation after surgery. Accordingly, greater emphasis has been placed on optimizing pancreaticojejunostomy techniques after Case 1 to prevent similar angular deformities. Nevertheless, the recurrent perforation observed in Case 2 alerts us that stent-induced intestinal perforation arises from the combined effects of multiple risk factors. Retrospective analysis of the surgical procedure in Case 2 suggests the following potential mechanisms: tension within the mesentery caused angulation of the afferent and efferent jejunal limbs at the hepaticojejunostomy site. Combined with postoperative edema at the hepaticojejunostomy that impeded the drainage of bile and pancreatic juice, the intestinal wall between the pancreaticojejunostomy and hepaticojejunostomy became edematous, thin, and less compliant. Therefore, stents with soft material and appropriate length should be selected to avoid intestinal wall irritation caused by overly rigid or excessively long stents. Additionally, the possibility of counterforce exerted by retrograde jejunal peristalsis as intestinal function recovers postoperatively also merits consideration.

We conducted a literature review focusing on complications secondary to pancreatic duct stent migration, among which jejunal perforation, intestinal obstruction, intestinal mucosal ulceration and hemorrhage are the most prevalent [14, 15]. Acute jejunal perforation is mainly characterized by severe abdominal pain accompanied by high fever and peritonitis; injury to the mesenteric vessels of the jejunum primarily manifests as intraperitoneal hemorrhage [5, 7, 9, 15]. Its occurrence is mainly associated with the following factors: (1) Excessively long indwelling segment of the stent within the jejunum and rigid stent material [5–7]. (2) Intestinal wall edema [5, 7]. (3) Angulation and torsion of the proximal jejunum induced by mesenteric tension [7]. (4) Internal stents soaked by bile, intestinal fluid and pancreatic juice are prone to colonization by intestinal bacteria and calcium salt deposition, which increases stent rigidity [6].

We performed a literature search in the PubMed database using the search terms and search strings as follows: “pancreatic duct stent migration”, “pancreaticojejunostomy stent”, “pancreaticoduodenectomy and stent migration”, “pancreaticoduodenectomy and jejunal perforation”, “pancreaticoduodenectomy and stent”, and “pancreaticojejunostomy stent migration”. The search period covered the database inception up to June 1, 2026. The literature inclusion criteria were set as follows: (1) Patients who underwent PD or LPD for benign or malignant diseases with intraoperative placement of internal pancreatic duct stents; (2) Cases with radiological, endoscopic or intraoperative evidence of intestinal complications attributed to pancreatic duct stents; (3) Complete diagnostic records and clear time intervals between surgery and complications.Exclusion criteria included studies with severely missing baseline patient data or duplicate reported cases. A total of 15 matching cases were retrieved for systematic analysis covering multiple variables: preoperative diagnosis, time interval from surgery to perforation, diagnostic methods for perforation, imaging findings, therapeutic strategies and long-term prognosis. Among the 15 patients, 12 received abdominal CT scans at the onset of clinical deterioration to confirm the diagnosis, which predominantly revealed abdominopelvic fluid collection. Except for stents that completely dislodged into the peritoneal cavity or penetrated deep through the intestinal wall, most stents penetrating the jejunal wall could hardly be identified on CT images [12]. This complication requires differential diagnosis from pancreatic fistula, biliary fistula and other postoperative adverse events in the early postoperative stage. Both patients in our center had brown-green abdominal drainage fluid after surgery. In the absence of acute abdominal pain, it is difficult to distinguish this condition from pancreatic or biliary fistula merely by the gross appearance of drainage fluid. Misdiagnosis may lead to severe intra-abdominal infection and septic shock, which can be life-threatening.

All 15 enrolled cases had complete treatment records. Among them, 9 cases (60%) developed perforation within one month after surgery, while 6 cases (40%) suffered perforation between one month and several years postoperatively. A total of 11 patients (73.7%) received reoperation, and jejunal repair was the predominant surgical procedure, accounting for 81.8% of reoperative cases. Three patients (20%) underwent endoscopic intervention, and one patient (6.6%) received interventional embolization due to intra-abdominal hemorrhage. The management strategy of migrated pancreatic duct stents was correlated with the interval from initial surgery to stent displacement and clinical manifestations. Surgical intervention was the primary option for patients presenting within one month postoperatively. By contrast, endoscopic stent retrieval was preferred for patients without acute abdomen whose complications occurred more than one year after the primary operation. All 15 patients recovered uneventfully and were discharged.

### Diagnostic and management algorithm for stent migration-induced jejunal perforation

**Figure:**
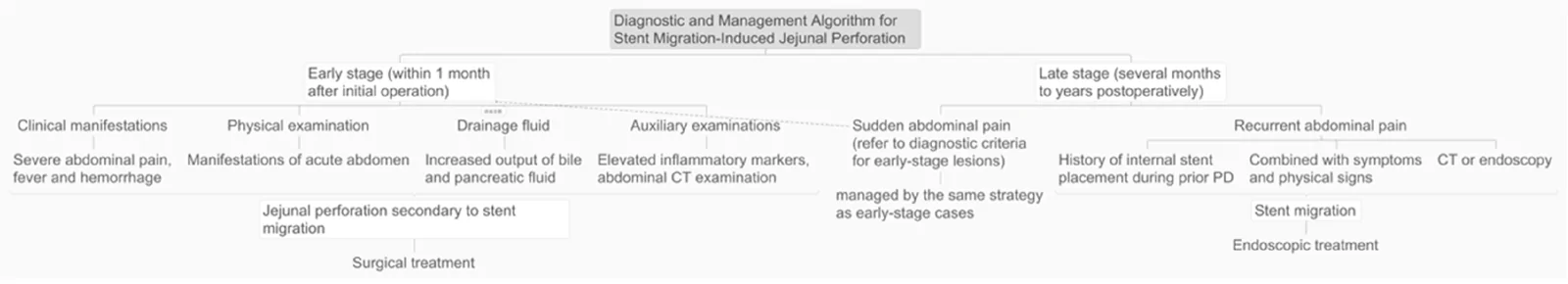

To prevent the occurrence of early perforation, soft silicone stents with an appropriate length of 6–8 cm that conform to the physiological length of the pancreatic duct should be selected, and overly long stents are not recommended. The jejunal tip of the stent should be spherical without sharp edges. The stent should be placed at a non-perpendicular angle and securely fixed with fewer than five interrupted sutures, with a diameter less than 5 Fr. This specification can prevent long-term complications secondary to delayed stent dislodgement caused by excessive sutures, oversized diameter or excessive stent length. Vital signs as well as the volume and characteristics of drainage fluid should be closely monitored postoperatively. Regular clinical follow-up is required after discharge. If the internal stent is not spontaneously expelled or becomes malpositioned one year after surgery, prophylactic endoscopic removal of the stent is advised to avoid subsequent long-term complications.

## Conclusions

Jejunal perforation caused by migrated pancreatic duct stents following pancreaticoduodenectomy is categorized into early and late phases. Early perforation mostly occurs within one month after surgery, whereas late perforation generally presents more than one year postoperatively. Severe abdominal pain, fever, hematemesis or intra-abdominal hemorrhage are common clinical manifestations in the early phase, while recurrent abdominal discomfort is the predominant symptom of late perforation. Reoperation is required for the majority of early-stage patients, and endoscopic intervention is the preferred treatment for late-stage cases.

### Patient perspective

For both Case 1 and Case 2, the patients were satisfied with the overall treatment process.

### Limitations

This study has several limitations. The cases were collected from a single center with a limited sample size. Multicenter studies with larger cohorts are needed to further verify our findings and draw more definitive conclusions.

## Supplementary Information

Supplementary Material 1. [5, 7, 12–17].

## Data Availability

No datasets were generated or analysed during the current study.

## Abbreviations

PD: Pancreaticoduodenectomy
LPD: Laparoscopic Pancreaticoduodenectomy
CT: Computed tomography
CECT: Contrast-Enhanced Computed Tomography
MRCP: magnetic resonance cholangiopancreatography
DSA: Digital subtraction angiography
ERCP: Endoscopic retrograde cholangiopancreatography
PNEP: Pancreatic neuroendocrine neoplasm
AA: Ampullary adenocarcinoma
PEP: Painful ectopic pancreas
PAC: Periampullary carcinoma
PA: Pancreatic adenocarcinoma
DPC: Duodenal papillary carcinoma
AC: Ampullary carcinoma
PHC: Pancreatic head carcinoma
DC: Duodenal carcinoma
CCA: Cholangiocarcinoma
