# Supplementary material for: Pancreatic stent migration-induced jejunal perforation following pancreaticoduodenectomy: case report and literature review
Source: BMC Surg. 2026 Aug 12;26:759. doi: 10.1186/s12893-026-04120-7 (PMC13746601; doi:10.1186/s12893-026-04120-7)
Supplement: Supplementary file 1 [file 12893_2026_4120_MOESM1_ESM.docx]

Clinical data of 15 patients with intestinal perforation induced by pancreatic duct stent migration

| **Case ID** | **Publication year / Author** | **Gender** | **Age** | **Preoperative diagnosis** | **Stent anastomosis method** | **Stent material** | **Time from surgery to perforation** | **Clinical manifestations** | **Treatment regimen** | **Prognosis** |
| --- | --- | --- | --- | --- | --- | --- | --- | --- | --- | --- |
| 1 | 2000/Biffl[14] | man | 78 | PENT | — | — | 2.5 years | Abdominal pain, abdominal distension, vomiting and cessation of defecation | Enterotomy for stent removal | Discharged |
| 2 | 2010/Layec[16] | female | 46 | AA | — | 12cm | 6 months | Recurrent abdominal and lumbodorsal pain | Stent retrieval via double-balloon enteroscopy | The patient maintained favorable survival status during 1-year follow-up |
| 3 | 2010/Layec[16] | man | 30 | PEP | — | 20cm | Several months | abdominal pain | Stent retrieval via double-balloon enteroscopy | The patient maintained favorable survival status during 1-year follow-up |
| 4 | 2015/Mari[12] | man | 53 | PAC | duct-to-mucosa anastomosis | 7 cm long 6 Fr Nelaton stent | 23 months | Severe abdominal pain with fever | Resection of the affected intestinal segment combined with stent removal | Discharged |
| 5 | 2018/Munis[13] | female | 69 | PA | — | — | 7 years | Severe lumbodorsal pain accompanied by fever | L3-L4 discectomy combined with paravertebral abscess debridement. Enterospinal fistula was suspected intraoperatively, subsequent endoscopic treatment failed, and laparotomy was performed for stent removal and jejunal repair | Discharged |
| 6 | 2020/Bao[7] | female | 71 | DPC | duct-to-mucosa anastomosis | a 15 cm free- oating Nelaton 7 Fr stent | 4 days | Severe abdominal pain with fever and 400 mL of brown-green abdominal drainage fluid | Trimming redundant stent segment, jejunal repair, and peritoneal irrigation with double-cannula drainage | The patient maintained favorable survival status during 1-year follow-up |
| 7 | 2023/Liu[15] | man | 76 | DPC | — | — | 6 days | A sharp increase in drainage fluid volume was observed shortly after surgery, with the amylase level of drainage fluid reaching 96400 U/L. The patient simultaneously presented with peritoneal irritation signs, severe abdominal pain and intra-abdominal hemorrhage | External stent drainage and hemostasis | Discharged |
| 8 | 2023/Liu[15] | man | 77 | AC | — | — | 15 days | The volume of postoperative drainage fluid increased rapidly within a short period, with drainage amylase up to 100000 U/L. Intra-abdominal hemorrhage occurred, accompanied by 800 mL bloody drainage fluid. | Digital subtraction angiography (DSA) arterial embolization; adjustment of stent position via ERCP | Discharged |
| 9 | 2023/Liu[15] | man | 67 | PHC | — | — | 10 days | Hematemesis of 300 mL | Celiac arteriography combined with jejunal mesenteric artery embolization | Discharged |
| 10 | 2025/Zou[5] | female | 59 | PHC | duct-to-mucosa anastomosis | 8 Fr silicone tube | 10 days | Abdominal pain accompanied by fever, with drainage fluid amylase level of 58450 U/L | Stent extraction plus jejunal repair | The patient achieved a good survival status during the 6-month follow-up period |
| 11 | 2026/Tamura[17] | female | 69 | PHC | duct-to-mucosa anastomosis | 6Fr internal pancreatic  stent (MD-41520, SB-KAWASUMI, Kanagawa Japan) | 36 days | Intra-abdominal hematoma | Stent removal and jejunal repair | Discharged |
| 12 | 2026/Tamura[17] | man | 83 | PHC | duct-to-mucosa anastomosis | 5Fr internal pancreatic  stent (MD-41515, SB-KAWASUMI, Kanagawa Japan) | 3 days | Active bleeding from the drainage tube | Trimming redundant stent and jejunal repair | Discharged |
| 13 | 2026/Tamura[17] | man | 66 | DC | duct-to-mucosa anastomosis | 6Fr internal pancreatic stent  (MD-41520, SB-KAWASUMI, Kanagawa Japan) | 5 days | Laboratory examinations indicated elevated systemic inflammatory response markers | Stent removal and jejunal repair | Discharged |
| 14 | case 1 | female | 57 | CCA | The suture of pancreaticojejunostomy was wrapped around the stent for one circle and knotted for fixation | 6 Fr 8 cm small-bore silicone drainage tube | 3 days | Severe abdominal pain combined with fever, with 500 mL brown-green fluid drained from the abdominal drainage tube | Trimming of redundant stent segment, jejunal repair, and peritoneal double-cannula irrigation drainage | The patient maintained favorable survival during the 48-month follow-up |
| 15 | case 2 | man | 50 | CCA | The suture of pancreaticojejunostomy was wrapped around the stent for one circle and knotted for fixation | 6 Fr 10 cm small-bore silicone drainage tube | 6 days | Severe abdominal pain combined with fever, with 400 mL brown-green fluid drained from the abdominal drainage tube | Trimming of redundant stent segment, jejunal repair, and peritoneal double-cannula irrigation drainage | The patient maintained favorable survival during the 37-month follow-up |
